# Psychometric evaluation of Persian version of the balanced measure of psychological needs scale among university students

**DOI:** 10.1002/nop2.854

**Published:** 2021-04-02

**Authors:** Hamid Sharif Nia, Pardis Rahmatpour, Fatemeh Khoshnavay Fomani, Gokmen Arslan, Omolhoda Kaveh, Saeed Pahlevan Sharif, Harpaljit Kaur

**Affiliations:** ^1^ School of Nursing and Midwifery Amol Mazandaran University of Medical Sciences Sari Iran; ^2^ Department of Nursing Alborz University of Medical Sciences Karaj Iran; ^3^ School of Nursing and Midwifery Tehran University of Medical Sciences Tehran Iran; ^4^ Department of Psychological Counseling and Guidance, Faculty of Education Burdur Mehmet Akif Ersoy University Burdur Turkey; ^5^ School of Nursing and Midwifery Sari Mazandaran University of Medical Sciences Sari Iran; ^6^ Taylor’s Business School Taylor’s University Lakeside Campus Subang Jaya Malaysia

**Keywords:** Balanced Measure of Psychological Needs, reliability, University Students, validity

## Abstract

**Aim:**

The purpose of the present study was to evaluate the reliability, validity and factor structure of the Persian version of the BMPN in Iranian university students.

**Design:**

Cross‐sectional.

**Methods:**

Study was conducted among Iranian medical sciences students from April to May 2020. A total of 660 students participated in the online self‐administrated questionnaire. Construct validity, convergent and divergent validity, and reliability of P‐BMPN were evaluated.

**Results:**

The Exploratory factor analysis showed that the Persian version of the BMPN has 17 items with four factors: dissatisfaction, autonomy Satisfaction, relatedness satisfaction and competence satisfaction that explained 40.17% of the total variance. Based on confirmatory factor analysis, all goodness‐of‐fit indices confirmed the model fit.

**Conclusion:**

These results suggest that the Persian version of the BMPN is a reliable and valid measure to assess satisfaction and dissatisfaction of the psychological needs in Iranian university students.

## 1 INTRODUCTION

It is important to acknowledge that human needs play crucial roles in driving peoples’ actions, which is the incitement of human behaviour. Maslow's Hierarchy of Needs, a well‐known motivation theory, states five categories of human needs that determines the behaviour of an individual.

The theory emphasizes on physiological, safety, love and belonging, esteem, and self‐actualization needs (Maslow & Lewis, 1987). In recent years, Maslow's theory has been criticized for its limitation regarding the generalizability of hierarchical pattern of needs, and lack of direct cause and effect relationship between need and behaviours (Fallatah & Syed, 2018; Trigg, 2004). To enhance the theory of human motivation, numerous theories have been developed over the years. William Glasser formulated the Theory of Choice and Reality Approach in 1998 and presented five basic human needs: survival, power, love, entertainment and freedom (Glasser, 1999). One of the most influential theoretical frameworks for studying motivation and social psychological processes is Self‐Determination Theory (SDT) (Neubauer & Voss, 2016).

The SDT is a macro‐organismic theory of human motivation and personality that considers the tendency of people's inherent growth and innate psychological needs (Deci et al., 1994; Gagné & Deci, 2005). Self‐determination theory grew out of the work of psychologists Edward Deci and Richard Ryan, who first introduced their ideas in their 1985 book, *Self‐Determination and Intrinsic Motivation in Human Behavior* (Deci & Ryan, 1985). This theory focuses on the degree to which an individual's behaviour is self‐motivated and self‐determined, and suggests that people are motivated to grow and change by three innate and universal psychological needs (Deci & Ryan, 2012). The determining factors in helping people to develop greater integrity and well‐being, and to achieve psychological nutriment are the sense of emotionally connected to others within warm, supportive and caring interpersonal relations (relatedness) (Baumeister & Leary, 1995), sense of self‐endorsement, volition and choice in the initiation and regulation of behaviour (autonomy), and experiences of mastery and effectance^1^ (competence) (Deci & Ryan, 1985, 2012; Ryan et al., 2008).

It is vital for individuals who are oriented towards achieving the psychological growth addressed by self‐determination theory, to maintain a continual sustenance as this growth does not happened instinctively (Ryan & Deci, 2017). Accordingly, the individual tendency to be either motivated or to remain passive is mainly affected by the individual's characteristics, such as the individual genetic makeup (Briley et al., 2018), and social conditions (Ryan et al., 2008). Furthermore, individual behaviour is very complex and a single source of motivation may not always be the underlying basis of the people's reactions (Li, 2019a,2019b; Tekçe et al., 2016) . Among the two main types of motivations that are addressed by SDT, individuals who are intrinsically motivated tend to engage in behaviours that are appealing to them to gain independence or knowledge without expecting any obvious external rewards. In contrary, extrinsic motivation occurs when the individuals are motivated to perform a behaviour or engage in an activity to earn a reward or avoid punishment (Tranquillo & Stecker, 2016). These two main motivation categories are the principal sources which individuals often draw on achieving their goals. The difference between intrinsic and extrinsic motivation is that extrinsic motivation arises from outside of the individual while intrinsic motivation arises from within (Deci & Ryan, 1985; Di Domenico & Ryan, 2017).

The needs are not only descriptive, indicative terms, but also imperative and deontic, and therefore must always be deconstructed based on personal goals, priorities and desires (Flaker, 2019). The human needs in SDT are based more on psychological basis and are defined as being the internal psychological factors required for psychological development, integration and well‐being. Accordingly, evidence from SDT revealed that satisfaction of innate psychological needs for autonomy, competence and relatedness play an important role as a mediator to foster well‐being (Deci & Ryan, 2000; Weinstein & Ryan, 2010). Despite the known positive correlation between well‐being and satisfying psychological needs, developing a valid measure to integrate three innate psychological needs is difficult, due to the complexity of need fulfilment operational meaning (Neubauer & Voss, 2016). This led to the development of the Basic Psychological Need Scale (BPNS) by Gagné in 2003 which assess the degree to which those three basics psychological need are fulfilled. Despite the importance of this measure regarding the psychological need, some limitation were identified in the concept analysis throughout the item generation (Schutte et al., 2018). BPNS was developed to assess 21 items: competence (6 items), autonomy (7 items) and relatedness (8 items). The subscale scores indicate the extent to which each individual's need is satisfied, while the total score signifies a general index of need satisfaction. However, past studies have revealed that the 21‐item three‐factor model did not sufficiently fit the data (Johnston & Finney, 2010; Schutte et al., 2018; Sheldon & Hilpert, 2012).

In response to the limitations in BPNS, Sheldon and Hilpert developed an alternative measure entitled Balanced Measure of Psychological Needs (BMPN) scale in 2012. BMPN is a 18‐ items measure that assess the level of psychological needs’ satisfaction/dissatisfaction and this scale has been employed in various studies, allowing its psychometric property to be identified (Cordeiro et al., 2016; Galiana et al., 2016; Neubauer & Voss, 2016). Considering the BMPN exclusivity to assess either overall fulfilment of the three psychological needs, or need satisfaction/dissatisfaction separately (Sheldon & Hilpert, 2012), the scale is identified as the most matched tool with SDT for assessing the basic psychological needs (Neubauer & Voss, 2016). In order to improve the model fit, Sheldon and Hilpert (2012) recommended that the three needs should be assessed separately instead of combining them into one overall need score (Neubauer & Voss, 2016; Sheldon & Hilpert, 2012).

Psychological need fulfilment is investigated in numerous studies, and its positive roles have been proven for improving well‐being (Tang et al., 2020), academic achievement (Tang et al., 2020), emotion regulation (Benita et al., 2020) and health‐related quality of life (Campbell et al., 2019). Based on past studies on different disciplines and professions, it was found that improving medical students' well‐being and quality of life is considered as a critical aspect of each health promotion program. Medical students need to be prepared for their medical careers which involves providing optimal care of patients, or establishing therapeutic relationship with patients and their families and therefore they need to pose well‐being (IsHak et al., 2013) that enhances their personal growth and self‐regulation capacity (Gagnon et al., 2016). To date, several studies have determined that the perceptions of medical students on psychological need satisfaction (autonomy, competence and relatedness) have enhanced sense‐making mentor‐student relationship (Janssen et al., 2020), improve their academic achievements (Feri et al., 2016), impacted their career decision autonomy, and career search behaviour (Lee & Kim, 2017) and protected them from burnout (Cho & Jeon, 2019; Ljubin‐Golub et al., 2020).

The existing body of research on the health status of Iran medical students has revealed the undesirable quality of life (Raiisi et al., 2020; Ziapour & Kianipour, 2018) due to the confrontation between satisfying individual needs and responding to university demands in clinical settings (Aliafsari Mamaghani et al., 2018), ambiguity in their medical role, and uncritical and dependent thinking climate (Yousefy et al., 2015). Medical students’ academic motivation is affected by several personal and organizational factors (Kunanitthaworn et al., 2018) in which autonomy (Bronson, 2016; Feri et al., 2016), clinical learning environment (Karabulut et al., 2015) and supportive relationship (Amit‐Aharon et al., 2020) are considered as important. To date, very little is known about the Iranian medical students' psychological needs fulfilment. Due to the important role of psychological needs satisfaction in psychological and physical health, unique features of the BMPN are being discussed. No previous study has investigated the Persian version of this scale; therefore, this study seeks to evaluate the reliability, validity and factor structure of the Persian version of the BMPN (P‐BMPNS) on Iranian university students.

## 2 METHODS

### 2.1 Design

This methodological study was conducted among Iranian medical sciences students from April to May 2020. The protocol of this study was approved by the Mazandaran University of Medical Sciences Research Ethics Committee (IR.MAZUMS.REC.1399.9073).

### 2.2 Study sample

The bench mark for sample size suggested by Kellar to conduct a factor analysis is between 5–10 samples per item of the intended instrument (Ebadi et al., 2012). In the current study, two independent samples were collected: 330 for exploratory factor analysis (EFA) and another 330 sample to evaluate the confirmatory factor analysis (CFA). In total, the data for 660 students were collected using online self‐administrated questionnaires. The questionnaire was designed on the Google Form platform, and its link was sent to the students of the country's universities through WhatsApp, Telegram and Gmail. And they were asked to send the link to their friends if they wished. The inclusion criteria for the respondents were that their participation was voluntary.

### 2.3 Measurements

The questionnaire comprised of socio‐demographic questions (age, gender and marital status), educational related information (type of university, field of study and GPA) and the Persian version of the BMPN scale.

The BMPN scale was developed by Sheldon and Hilpert in 2012. It consists of 18 items encompassing three factors: competence (6 items), autonomy (6 items) and relatedness (6 items). The BMPN scale is scored on a 5‐point Likert‐type scale from 1 (never)–5 (always). The odd items are negative (dissatisfaction), and even items are positive (satisfaction). The internal consistency values for the three‐dimensional forms of autonomy, competence and relatedness models were found to be 0.68, 0.75 and 0.84, respectively (Sheldon & Hilpert, 2012).

### 2.4 Translation

Written permission for the use of the BMPN scale was obtained from the developer, Dr. Kennon M. Sheldon. The World Health Organization's (2016) protocol of forward–backward translation technique was applied to translate the scale from English into Persian. Two English‐Persian translators were asked to independently translate the questionnaire. Subsequently, a Persian‐English translator was invited to back‐translate the Persian scale to English.

### 2.5 Statistical analyses

#### 2.5.1 Content validity

Content Validity Ratio (CVR) and Content Validity Index (CVI) were calculated by 10 faculty members in the medical education and psychology. When the number of experts is 10, the minimum acceptable CVR based on Lawshe's table is 0.62 (Lawshe, 1975). The minimum acceptable value for CVI for each item is 0.7.

#### 2.5.2 Construct validity

Construct validity was evaluated through the maximum‐likelihood exploratory factor analysis (MLEFA) with Promax rotation. The Kaiser–Meyer–Olkin test (KMO) and Bartlett's test of sphericity were used to check the appropriateness of the study sample and the model. The numbers of factors were determined based on parallel analysis and exploratory graph analysis. Items with absolute loading values of at least 0.3 were considered appropriate (Çokluk & Koçak, 2016).

The presence of an item in a latent factor was determined based on a factor loading of almost 0.3, which was estimated using the following formula: CV = 5.152 ÷ √ (*n* – 2), where CV was the number of extractable factors and “n” was the sample size. The numbers of latent factors were estimated using Horn's parallel analysis and items with communalities <0.2 were excluded from the EFA. For assessment of the structural factors, a confirmatory factor analysis (CFA) was conducted using the maximum‐likelihood method and the most common goodness‐of‐fit indices. The model fitness was assessed by six indices: Root Mean Square of Error of Approximation (RMSEA), Comparative Fit Index (CFI), Parsimonious Comparative Fit Index (PCFI), Parsimonious Normed Fit Index (PNFI), Incremental Fit Index (IFI) and CMIN/DF. In CFA, items with standardized factor loading lower than 0.5 were removed from the model.

#### 2.5.3 Convergent and divergent validity assessment

The convergent and divergent validity of the Persian version of the BMPN scale were estimated using Fornell and Larcker's approach. The average Variance Extracted (AVE) and Maximum Shared Squared Variance (MSV) were estimated to assess the convergent and discriminant validity of the extracted factors. To establish convergent validity, the AVE and Composite Reliability (CR) values should be greater than 0.5 and 0.7, respectively. In order to achieve the discriminant validity criteria, the MSV of each construct should be less than its AVE (Ahadzadeh et al., 2015).

#### 2.5.4 Reliability assessment

Internal consistency reliability was assessed by employing Cronbach's alpha (α), McDonald's omega (Ω) and Average Inter‐item Correlation (AIC). The values of the coefficients Ω and α which were greater than 0.7 were acceptable (Mayers, 2013) whereas the AIC value between 0.2–0.4 indicated good internal consistency (Hackman et al., 2006). CR, which replaces Cronbach's alpha coefficient in structural equation modelling, was then evaluated, and a CR value greater than 0.7 was considered acceptable (Sharif Nia et al., 2019).

#### 2.5.5 Multivariate normality and outliers

Univariate and multivariate distributions were examined for outliers by skewness, and kurtosis and the Mardia's coefficient, respectively. One indication of deviation from normal distribution is a Mardia's coefficient >8. Multivariate outliers were evaluated through the evaluation of a Mahalanobis distance. Items with a Mahalanobis distance of *p* < .001 were considered to be multivariate outliers (Esposito Vinzi et al., 2010). All of the statistical analyses were performed using SPSS‐AMOS, SPSS R‐Menu and JASP.

## 3 RESULTS

In this study, majority of the students were female (70.3%) and single (89.4%). The mean age and GPA of participants were 22.70 years old (*SD* = 5.2) and 16.57 of 20 (*SD* = 1.9), respectively.

**FIGURE 1:**
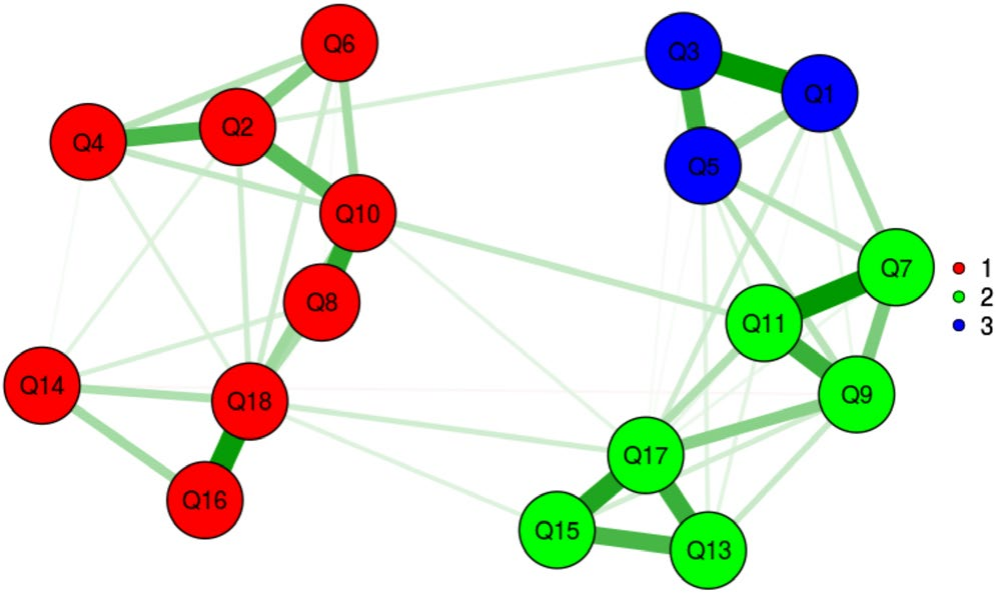
Exploratory Graph Analysis (EGA)

**FIGURE 2:**
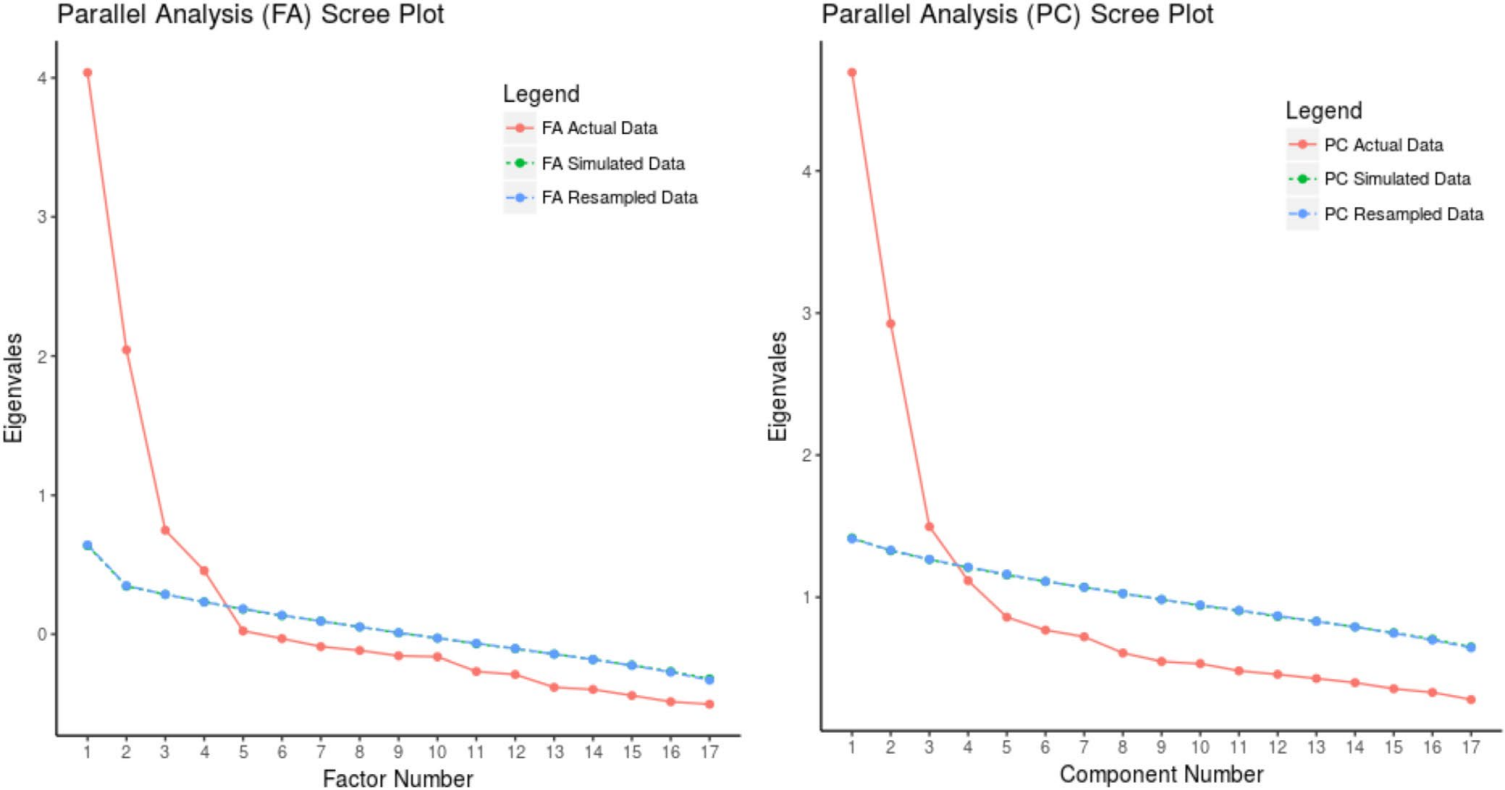
. Pararll analysis scree plot

In the MLEFA, the KMO test value was 0.902 and Bartlett's test value was 2638.649 (*p* <.001). The MLEFA revealed a four‐factor structure of scale namely dissatisfaction, autonomy satisfaction, competence satisfaction and relatedness satisfaction (Figures 1 and 2).

**FIGURE 3:**
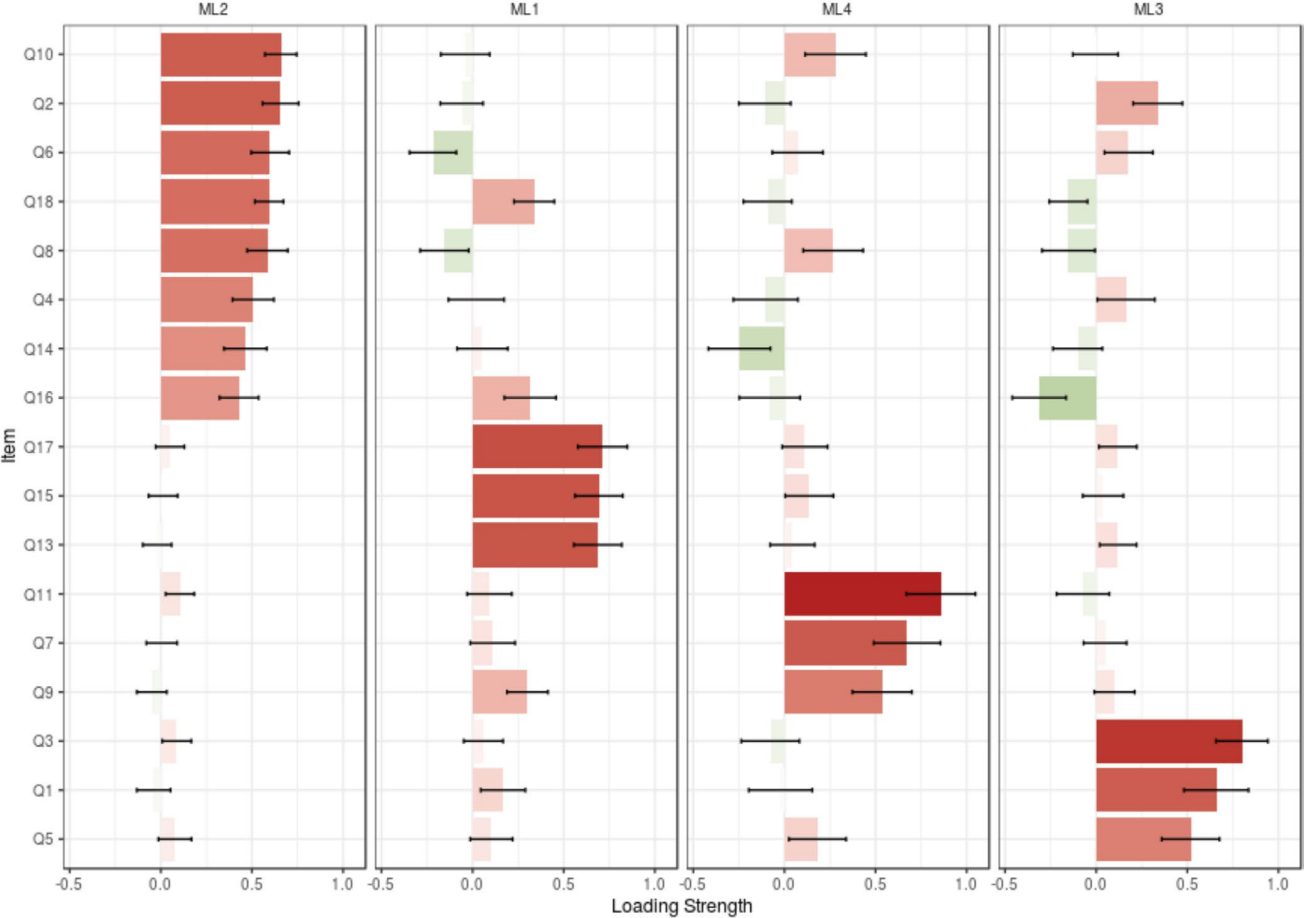
Loading strength of extracted factors

These four factors (17 items) explained 40.17% of the total variance of the Persian version of the BMPN among the Iranian University students. The eigenvalues and extracted variance of each factor were provided in Table 1. Also, see Figure 3 that indicated the loading strength of each items.

**TABLE 1:** Exploratory factors extracted from 17 items of the Persian version of the BMPN

Factor	Q_n_. Item	Factor loading	h^2^	Eigenvalue	% Variance
Dissatisfaction	10 I did something stupid, that made me feel incompetent.	0.664	0.515	2.59	15.24
	2 I was lonely.	0.657	0.494		
	6 I had disagreements or conflicts with people I usually get along with.	0.603	0.376		
	18 I had to do things against my will.	0.595	0.565		
	8 I experienced some kind of failure, or was unable to do well at something	0.586	0.372		
	4. I felt unappreciated by one or more important people.	0.513	0.283		
	14. I had a lot of pressures I could do without.	0.459	0.300		
	16. There were people telling me what I had to do.	0.430	0.410		
Autonomy satisfaction	17. I was really doing what interests me.	0.711	0.650	1.47	8.64
	15. My choices expressed my “true self”.	0.703	0.575		
	13. I was free to do things my own way.	0.683	0.526		
Competence satisfaction	11. I did well even at the hard things.	0.845	0.736	1.44	8.47
	7. I was successfully completing difficult tasks and projects.	0.669	0.535		
	9. I took on and mastered hard challenges.	0.526	0.555		
Relatedness satisfaction	3. I felt close and connected with other people who are important to me.	0.793	0.594	1.33	7.82
	1. I felt a sense of contact with people who care for me, and whom I care for.	0.654	0.487		
	5. I felt a strong sense of intimacy with the people I spent time with.	0.523	0.447		

CFA results confirmed the final model with 17 items. After modification, all goodness‐of‐fit indices confirmed the model fit as shown in Table 2.

**TABLE 2:** Fit model indices of the CFA of the Persian version of the BMPN (*n* = 330)

CFA model	286.444	111	<0.001	2.581	0.069	0.709	0.746	0.916	0.915
Indices Model	χ^2^	Df	*p* value	CMIN/DF	RMSEA	PCFI	PNFI	IFI	CFI

**FIGURE 4:**
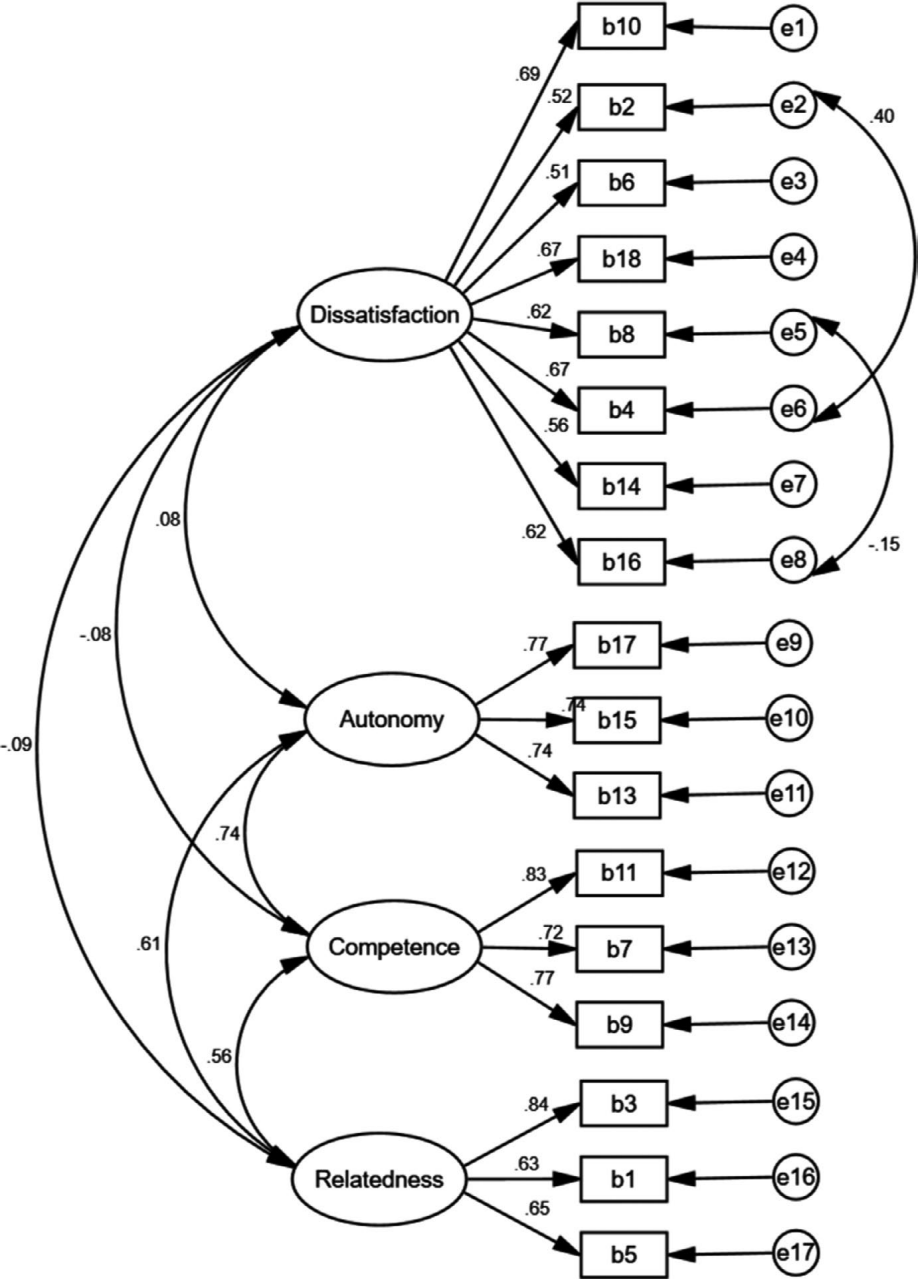
The Persian version of the BMPN construct: modified model of confirmatory factor analysis (*n* = 330)

Convergent and divergent validity of the Persian version of the BMPN scale were evaluated and the AVE of factors was greater than MSV in all the factors, indicating that the factors have good convergent and divergent validity (refer to Table 3 and Figure 4). Cronbach's alpha, McDonald's omega and CR of the four extracted factors were good, and the AIC values of the factors indicated good internal consistency (refer to Table 3).

**TABLE 3:** Convergent, divergent validity and composite reliability indices of the Persian version of the BMPN

Factors	CR	AVE	MSV	Max(R)	Alpha [CI 95%]	Omega	AIC
Dissatisfaction	0.819	0.365	0.009	0.831	0.78 [0.74–0.81]	0.80	0.315
Autonomy satisfaction	0.791	0.558	0.543	0.792	0.79 [0.75–0.83]	0.82	0.570
Relatedness satisfaction	0.752	0.506	0.377	0.787	0.80 [0.77–0.84]	0.82	0.586
Competence satisfaction	0.818	0.601	0.543	0.827	0.74 [0.69–0.78]	0.77	0.492

## 4 DISCUSSION

The results revealed a four‐factor structure of scale, comprising of 17 items, namely dissatisfaction (8 items), autonomy satisfaction (3 items), competence satisfaction (3 items) and relatedness satisfaction (3 items). These four factors explained 40.17% of the total variance of the BMPN among the Iranian university students. Based on the findings, Cronbach's alpha, McDonald's omega, CR and maximal reliability of four extracted factors of the BMPN were good. In addition, the internal consistency of subscales found in the present study was close to the original scale; dissatisfaction alphas was 0.78 ranging from [.74 to 0.81], autonomy satisfaction alphas was .79 ranging from [.75 to 0.83], relatedness satisfaction alphas was .80 ranging from [.77 to .84] and competence satisfaction alphas was. 74 ranging from [.69 to .78]; (Sheldon & Hilpert, 2012). The AIC values of factors were found to have good internal consistency (.49).

The results of the present study are consistent with studies done by Neubauer & Voss in 2016 using the German population (Neubauer & Voss, 2016) and Cordeiro et al. (2016) among Portuguese high school students (Cordeiro et al., 2016) who showed that fit index values of six‐dimensional model were better than three dimensions for Turkish Culture (Kardaş & Yalçin, 2018). Sheldon and Hilpert (2012) in the original development study of the scale reported measures of three with six dimensions models of needs in the satisfaction and dissatisfaction (Sheldon & Hilpert, 2012).

Based on the EFA results, the Persian version of BMPN consists of four factors which were very similar to the original scale. The first factor dissatisfaction, referred to feel incompetent, failure experience, disagreements or conflicts with the people, do things against will, felt unloved, high pressures, forcing others to do things. Based on the past literature, dissatisfaction may negatively affect well‐being and stimulate ill‐being and cause higher vulnerability for psychopathology especially among the young people (Hui et al., 2019; Kardaş & Yalçin, 2018). Cordeiro et al. (2016) believed that dissatisfaction of each need is related to the subjective experiences of low satisfaction of autonomy, competence and relatedness needs that isolates people, need dissatisfaction predicted anxiety, depression and somatization in an individual (Cordeiro et al., 2016). Therefore these factors are important implications for the measurement of the psychological needs.

The second factor of the P‐BMPN is “autonomy satisfaction,” which is related to doing what interests, show true self and do things freely. Multiple studies have shown that there exists a correlation between teachers’ autonomy support and academic performance (Gutiérrez et al., 2018; Jang et al., 2010; Li, 2019a, 2019b; Perry et al., 2010). In the same vein, Jang et al. (2012) research revealed that students’ perception of autonomy support influenced students’ experienced autonomy need satisfaction, and students’ engagement (Jang et al., 2012). Other studies found that autonomy satisfaction increased via motivational and meaningful activities in students, facilitates students’ autonomous self‐regulation for learning and the influence of trust and school engagement, have positive consequences in one's academic achievement (Bronson, 2016; Heyns & Rothmann, 2018). This connection has been supported by the findings of the current study. The role of BMPN on school engagement and academic success have previously been reported by a number of studies (Gutiérrez et al., 2018; Jang et al., 2010; Raufelder et al., 2014) but, however, based on the findings, the role of the teachers in satisfying the above need was very highlight and prominent.

The third factor of the P‐BMPN is “competent satisfaction,” which is related to do well even at the hard thing, successfully completing difficult tasks and mastered hard challenges. These factors, like other factors, have significant impact on the well‐being of students, their subjective vitality and their academic success. This accords with previous literature that confirms that teachers stimulate or reinforce feelings of competence on their students (Cordeiro et al., 2016; Galiana et al., 2016).

The last factor extracted is “relatedness satisfaction,” and it referred to feel close and connected with other people, sense of contact with people who care for me, and whom I care for and strong sense of intimacy with the people. Previous studies on relatedness satisfaction described it as warm, supportive and caring interpersonal relations (Baumeister & Leary, 1995; Ryan & Deci, 2000). It is vital to note which relatedness frustration is associated with the experience of relational rejection, loneliness and depression (Cho & Jeon, 2019; Cordeiro et al., 2016).

Individuals in different cultures vary in their perceptions of need satisfaction and well‐being, also the impact of culture on psychological needs is undeniable (Church et al., 2013). In this regard, Muslims beliefs and culture are different from other religion; thus, this study was conducted among Iranian university students.

In sum, autonomy and competence support via feeling listened to and understood and feeling valued and acknowledge promote and develop satisfaction of relatedness that lead to increase their motivation and well‐being (Lynch et al., 2005). Also, Kamel and Hashish (2015) recommended that organizations.

should enhancing a more positive and supportive work environment that improve their sharing, cooperating, learning and belonging in other words, satisfy the psychological needs of people (Kamel & Abou Hashish, 2015). For this reason, there is a need to pay special attention to the psychological needs of medical students who enter the clinical setting and hospital environment after graduation, as they need to provide high‐quality services to their patients. Satisfaction of needs and psychological well‐being are important and vital in the quality of life of the professionals and quality of their patient care.

### 4.1 Limitation

The first limitation for this study is the recall bias as the data were collected by self‐reported questionnaires online and therefore the generalizability should be done with caution. The next limitation was the use of convenience sampling which resulted in majority of the participants being female Iranian university students and their psychological needs are different since gender balance was not possible.

## 5 CONCLUSION

The results of the current study showed that the Persian version of the BMPN has good construct, convergent and divergent validity and reliability. The model fit values were appropriate. These findings of this study suggest that the Persian version of the BMPN is a reliable and valid measure to evaluate satisfaction and dissatisfaction of the psychological needs in terms of autonomy, competence and relatedness among Iranian university students.

## CONFLICT OF INTEREST

No conflict of interest has been declared by the authors.

## ETHICAL APPROVAL

The protocol of this study was approved by the Mazandaran University of Medical Sciences Research Ethics Committee (IR.MAZUMS.REC.1399.7523).

## Data Availability

The data that support the findings of this study are available on request from the corresponding author.

## ENDNOTE

^1^
The state of having a causal effect on objects and events in the environment; the word is commonly used in the term **effectance motivation**, coined by Robert W. White in 1959.
